# Cognitive Alterations in Motor Imagery Process after Left Hemispheric Ischemic Stroke

**DOI:** 10.1371/journal.pone.0042922

**Published:** 2012-08-09

**Authors:** Jing Yan, Xiaoli Guo, Zheng Jin, Junfeng Sun, Liwei Shen, Shanbao Tong

**Affiliations:** 1 School of Biomedical Engineering, Shanghai Jiao Tong University, Shanghai, China; 2 Department of Neurology, The Fifth People’s Hospital of Shanghai, Shanghai, China; 3 Med-X Research Institute, Shanghai Jiao Tong University, Shanghai, China; Hôpital Robert Debré, France

## Abstract

**Background:**

Motor imagery training is a promising rehabilitation strategy for stroke patients. However, few studies had focused on the neural mechanisms in time course of its cognitive process. This study investigated the cognitive alterations after left hemispheric ischemic stroke during motor imagery task.

**Methodology/Principal Findings:**

Eleven patients with ischemic stroke in left hemisphere and eleven age-matched control subjects participated in mental rotation task (MRT) of hand pictures. Behavior performance, event-related potential (ERP) and event-related (de)synchronization (ERD/ERS) in beta band were analyzed to investigate the cortical activation. We found that: (1) The response time increased with orientation angles in both groups, called “angle effect”, however, stoke patients’ responses were impaired with significantly longer response time and lower accuracy rate; (2) In early visual perceptual cognitive process, stroke patients showed hypo-activations in frontal and central brain areas in aspects of both P200 and ERD; (3) During mental rotation process, P300 amplitude in control subjects decreased while angle increased, called “amplitude modulation effect”, which was not observed in stroke patients. Spatially, patients showed significant lateralization of P300 with activation only in contralesional (right) parietal cortex while control subjects showed P300 in both parietal lobes. Stroke patients also showed an overall cortical hypo-activation of ERD during this sub-stage; (4) In the response sub-stage, control subjects showed higher ERD values with more activated cortical areas particularly in the right hemisphere while angle increased, named “angle effect”, which was not observed in stroke patients. In addition, stroke patients showed significant lower ERD for affected hand (right) response than that for unaffected hand.

**Conclusions/Significance:**

Cortical activation was altered differently in each cognitive sub-stage of motor imagery after left hemispheric ischemic stroke. These results will help to understand the underlying neural mechanisms of mental rotation following stroke and may shed light on rehabilitation based on motor imagery training.

## Introduction

Motor imagery is a cognitive process in which the representation of a specific motor action is internally reactivated within working memory but without an overt motor output. It is a motor program obeying the principles of central motor control [1]–[4]. Neuroimaging studies on health subjects showed that motor imagery mostly activated the same brain areas as in real movement execution [5]–[7]. Any injury, e.g., stroke, in motor-related brain areas could lead to prominent motor function impairment [8], [9]. Interestingly, recent studies on stroke patients with motor disability revealed that their motor imagery ability was still intact or partially preserved, which has been gaining attention in rehabilitation research of motor function recovery [10]–[14].

It was reported that stroke patients who received motor imagery training could improve the motor rehabilitation [10], [15]. Further study on patients of one-year post stroke showed significantly improved arm function after the combined physical therapy and motor imagery training compared with combined physical therapy and relaxation training [15]. Although motor imagery training has been methodologically well designed and the results have justified some optimism in stroke rehabilitation, the majority of these studies to date were poorly controlled case or with small sample sizes, and the outcomes were inconsistent [10], [11], [15]–[20]. Furthermore, the underlying neural mechanisms of motor rehabilitation by motor imagery have not been fully understood. The studies so far have been mostly based on behavior assessment and overall neural activations during motor imagery with few temporal details of the cognitive process. Therefore, one interesting question is how stroke lesion would affect the cortical activation pattern of each sub-stage during the cognitive task. In this paper, we aim to investigate the alterations in motor imagery cognitive process after stroke using high temporal resolution neural-electrophysiological EEG recordings.

In most experimental paradigms, motor imagery was measured using a mental rotation task (MRT) in which pictures (e.g., hands) were presented in different spatial orientations and participants were asked to make a laterality (e.g., left or right hand) judgment from the “first-person” perspective [4], [21]–[23]. Most studies showed that mental rotation was carried out similarly to the actual rotation of the body part and the response time (RT) increased with the rotation angle [24], [25]. It was proposed that longer RT was due to the fact that the picture had to be mentally rotated to the upright position for parity judgment [24]. The cognitive process of MRT has been considered with three sub-stages, i.e., (i) visual stimulus perceptual encoding (identification of stimulus and its orientation), (ii) mental rotation, and (iii) response [26]–[28]. For MRT of hand pictures, subjects compared the picture stimulus to a mental representation of their own hand through visuo-spatial and motor integration. Such a mental activation shared, at least partially, the same cognitive process of actual movement planning and execution [25], [29], [30]. Neuroimaging studies showed that cortical neural networks including posterior parietal and visual cortex, premotor cortex (BA6), supplementary motor areas (SMA) and primary motor cortex (M1) were activated during mental rotation of body parts [12], [30]–[33]. In addition, some subcortical structures, e.g., basal ganglia, were also activated during mental rotation [34]. Basal ganglia and motor cortices have been known to be involved in motor planning and execution, and their activation during MRT suggested that actual and mentally simulated movements share largely overlapping neural structures [7], [30], [31], [35]. Meanwhile, studies by electrophysiological measures, e.g., ERP, showed that MRT elicited two positive components at parietal area in 0–300 ms (P200) and 300–800 ms (P300) [27], [36], [37]. P200 was considered to be related to visual stimulus perceptual encoding, while P300 in MRT was a critical component and became relatively more negative with the increase of angle from the upright position, which was called “amplitude modulation effect” [27], [38]. Although ERP was a useful technology to show significant brain electrophysiological activations during motor imagery, it was only considered to be phase resetting of ongoing EEG oscillations evoked by external stimuli [39]. Actually, some late cognitive brain oscillations are not ideally phase-locked, which may thus be eliminated in ERP averages, whereas (de)synchronization (ERD/ERS) analysis could keep the phase-unlocked information [40]. ERD/ERS phenomena have been reported in a wide range of real motor tasks as well as motor imagery [41], [42]. Significant ERD in beta band over sensorimotor areas in movement task was thought to be related to movement preparation and execution, which was then followed by beta ERS over precentral cortex, reflecting an idling and deactivation of motor cortex [43]–[46]. In this study, combining behavior, ERP and ERD/ERS methods, we are going to investigate the alternations of cortical activation in each cognitive sub-stage during motor imagery task for the patients with ischemic stroke in left hemisphere.

## Materials and Methods

### Ethics Statement

This study was approved by the Ethics Committee of Shanghai Jiao Tong University and The Fifth People’s Hospital of Shanghai. The written consent form was signed by each participant before any study procedures were done. All clinical investigation was conducted according to the principles expressed in the Declaration of Helsinki.

### Subjects

Eleven patients (mean age 60.3±12.7 years, range: 45–80 years, male/female  = 9/2) with mild or moderate ischemic stroke in left hemisphere were recruited from the Department of Neurology in the Fifth People’s Hospital of Shanghai (demographic details in Table 1). The same number of age-matched control volunteer subjects (mean age 60.1±6.9 years, range: 45–68 years, male/female  = 6/5) were recruited from local community. All control subjects reported no history of seizures, neurological diseases, or psychiatric disorders. All participants were right-handed with normal or correct-to-normal vision and gave their written informed consent prior to their inclusion in this study.

**Table 1:** Demography of stroke patients.

Patients	Gender	Age (years)	Post-stroke (months)	NIHSS	Lesion location
P1	M	74	6	9	Parietal lobe, basal ganglia
P2	M	53	9	10	Frontal lobe
P3	M	45	8	8	Frontal, parietal lobe
P4	F	46	12	8	Parietal lobe
P5	M	66	4	7	Parietal lobe
P6	M	64	5	9	Frontal lobe, basal ganglia
P7	M	57	7	8	Frontal, parietal lobe
P8	F	55	6	6	Frontal lobe
P9	M	80	14	6	Frontal lobe, basal ganglia
P10	M	46	9	7	Frontal, parietal lobe
P11	M	77	10	8	Parietal lobe, basal ganglia
Average		60.3±12.8	8.2±3.0	7.8±1.2	

M =  Male.

F =  Female.

NIHSS  =  National Institute of Health Stroke Scale.

### Experiments

**Figure 1:**
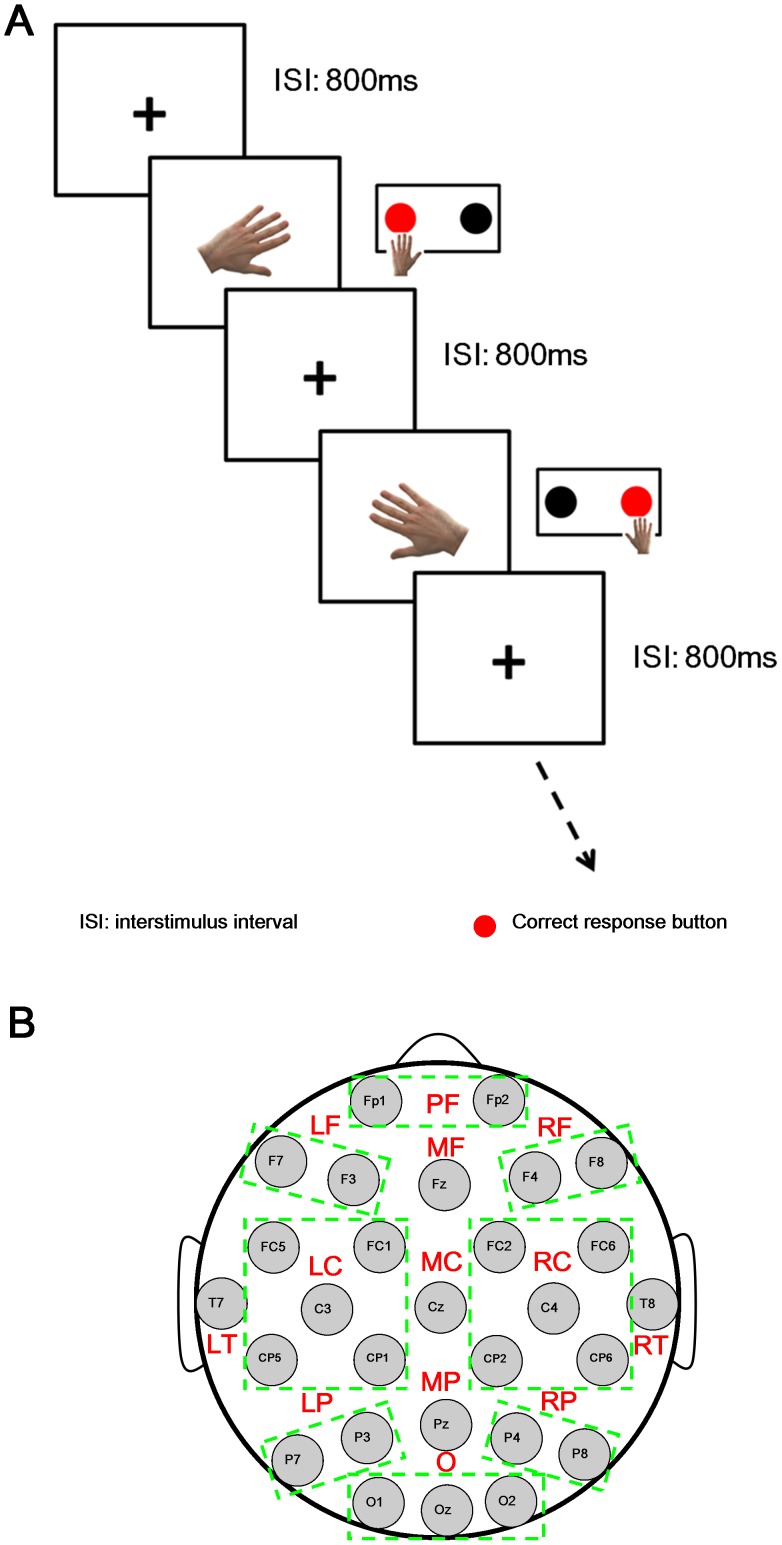
Schematic diagram of experimental procedure and topological cortical regions. **A:** Experimental procedure with red wafer indicated the correct response button was illustrated. ISI denoted interstimulus interval (i.e., 800 ms with black crosshair “+” in this study). **B:** Grouped thirteen cortical regions with their abbreviations, (i.e., PF (prefrontal area, Fp1 and Fp2), LF (left frontal area, F3 and F7), MF (middle frontal area, Fz), RF (right frontal area, F4 and F8), LT (left temporal area, T7), LC (left central area, FC1, FC5, CP1, CP5 and C3), MC (middle central area, Cz), RC (right central area, FC2, FC6, CP2, CP6 and C4), RT (right temporal area, T8), LP (left parietal area, P3 and P7), MP (middle parietal area, Pz), RP (right parietal area, P4 and P8), O (occipital area, O1, Oz and O2)) were shown.

Experimental paradigm was illustrated in Figure 1A. During experiment, stimulus pictures (size: 9 cm ×9 cm) of either right or left hand were randomly presented on the display with a viewing angle of approximate 2.5° in height. Both left and right hand pictures were presented at six different angles: 0°, 60°, 120°, 180°, 240° and 300°. Thus in total there were 12 (2×6: [HAND (left and right)] × [ANGLE (0°, 60°, 120°, 180°, 240° and 300°)]) stimulus types. Each experiment consisted of two blocks for the patient or six blocks for the control subject. In each block, there were 96 stimuli (left hand: right hand  = 50%:50%) with probabilities: 0°, 25%; 60°, 12.5%; 120°, 12.5%; 180°, 25%; 240°, 12.5%; 300°, 12.5%. A black cross symbol “+” was displayed during the 800 ms interstimulus interval (ISI). The pictures were presented until participants pressed the response button. All experimental parameters were chosen according to the data in the test trials prior to the experiment. Stimulation timing and the synchronization between behavioral and EEG data acquisition were controlled by E-Prime 2.0 (Psychology Software Tools, Inc, Pittsburgh, USA). Subjects were asked to keep minimal head and eye movements during the experiment. They were requested to press the left button using left index finger for left hand stimuli and the right button using right index finger for right hand stimuli as quickly and accurately as possible. There was a 3∼5 min inter-block break for resting.

### Behavior Analysis

[+2]Two behavior parameters (i.e., RT and accuracy rate (ACC)) were investigated in this study. ACC was the ratio of the correctly responded trials to the total trials by stimulus type. RT was the time from the onset of visual stimulus pictures to the button response by stimulus type also. Only correctly responded trials were considered in RT analysis.

### EEG Data Acquisition

EEG signals were continuously recorded using Vision Recorder (Version 1.03, Brain Products GmbH, Munich, Germany) from 32 channel Ag-AgCl electrodes (Fast’n Easy 32 Ch-Standard Cap, Brain Products GmbH, Munich, Germany) at a sampling rate of 1000 Hz and impedance below 5 kΩ at each electrode. An electrode between Fz and Cz (i.e., Fcz) served as the reference. EEG signals were offline re-referenced to an average of electrodes at left mastoid and right mastoid [47]. Horizontal and vertical electro-oculograms (EOGs) were also recorded for removing eye-movement artifacts by means of ICA method implemented in Analyzer software (Brain Products GmbH, Munich, Germany) [48]. After re-reference and EOGs removal steps, totally there were 28 channels of EEG signals in this study. Signals with artifacts greater than 100 µv or less than −100 µv were excluded (∼12.6% of all trials). EEG signals were then band-pass filtered into the range of 0.01–80 Hz for further data analysis.

### ERP Analysis

EEGs were segmented into trials of 200 ms baseline plus 1400 ms post stimulus onset. All trials in the same stimulus type with correct answer were grand-averaged for ERP analysis. Individual peak amplitude in two time windows, i.e., P200 (0 ms∼300 ms) and P300 (300 ms∼800 ms), was extracted for further statistical analysis of ERP. To investigate the topological patterns of the ERP activities, all 28 channels were grouped into thirteen regions according to their scalp locations: PF(prefrontal area, Fp1 and Fp2), LF(left frontal area, F3 and F7), MF(middle frontal area, Fz), RF(right frontal area, F4 and F8), LT(left temporal area, T7), LC(left central area, FC1, FC5, CP1, CP5 and C3), MC(middle central area, Cz), RC(right central area, FC2, FC6, CP2, CP6 and C4), RT(right temporal area, T8), LP(left parietal area, P3 and P7), MP(middle parietal area, Pz), RP(right parietal area, P4 and P8), O(occipital area, O1, Oz and O2) in the following statistical analysis [49] (see Figure 1B).

### ERD/ERS Analysis

All artifact-removed EEGs were then band-pass filtered into the beta band (14–30 Hz) for ERD/ERS analysis. Filtered EEGs were then segmented into trials from −600 ms to 1400 ms (for control subjects) or 4200 ms (for stroke patients) referring to the stimulus onset. All trials with correct response were squared and then smoothed by moving window average method (window size: 50 ms) to reduce the variance. After averaging the smoothed EEGs over trials by stimulus type, we obtained the ERD/ERS curve. In accordance with the cognitive process of MRT, ERD/ERS was then segmented into four sub-stages, i.e., Baseline (−600∼0 ms), Beginning (0∼300 ms, corresponding to P200), Middle (300∼800 ms, corresponding to P300) and End (800∼1500 ms for control group, 800∼4200 ms for patients group).Then four ERD/ERS indexes, corresponding to four sub-stages respectively, were obtained after averaging all ERD/ERS values in each sub-stage and normalization with Baseline. Topological patterns of ERD/ERS were compared and statistically analyzed according to the cortical regions (Figure 1B).

### Statistical Analysis

Repeated measures analysis of variance (ANOVA) was used to statistically analyze the behavior performances (ACC and RT), ERP components (amplitudes of P200 and P300) and beta-ERD/ERS indexes. In all analysis, GROUP (control vs. patients) was the between-subjects factor. And within-subjects factors were separately described in each result session. Statistical significance was accepted for values of p<0.05.

## Results

### Behavioral Results

**Figure 2:**
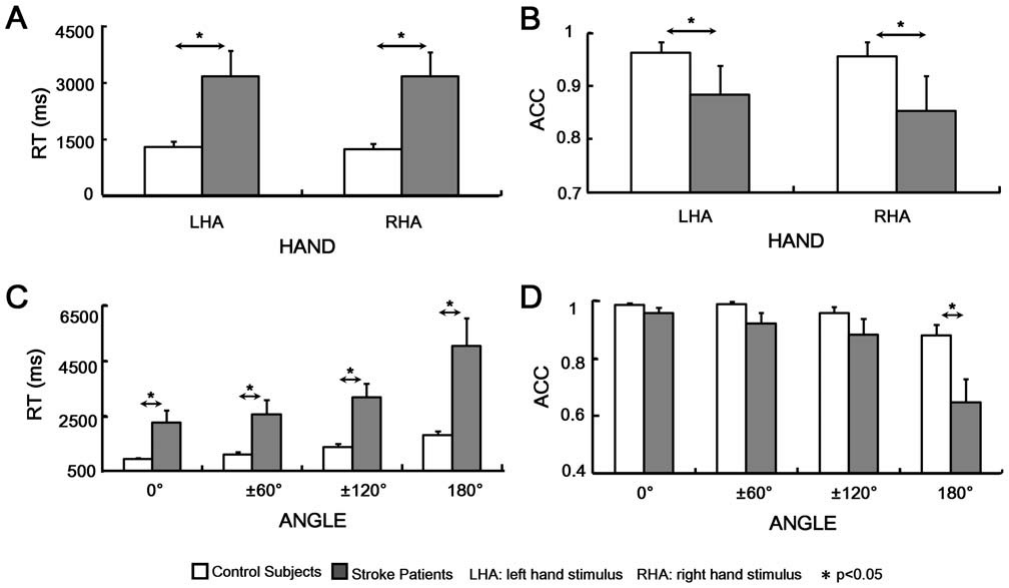
Behavior performance. **A** and **C:** Response time was illustrated in aspects of HAND and ANGLE factors respectively. **B** and **D:** Accuracy rate was shown also with respect to HAND and ANGLE factors respectively. White bar represented control subjects and gray bar represented stroke patients. Significance was indicated with*.

Two-way repeated measures ANOVA was performed on behavior data (i.e., ACC and RT). The within-subjects factors were HAND (left hand stimuli (LHA) vs. right hand stimuli (RHA)) and ANGLE (0° vs. 60° vs. 120° vs. 180° vs. 240° and 300°). ANOVA of RTs showed significant main effect of GROUP (F(1,20) = 14.419, p = 0.001), which was mainly due to longer response time in stroke patients (3167±491 ms) than control subjects (1270±88 ms). Consistently, stroke patients had significantly lower ACC than control subjects (patients vs. controls: 0.86±0.03 vs. 0.95±0.01; GROUP effect: F(1, 20) = 6.871, p = 0.017) (Figure 2). The insignificance of the HAND effect on RTs and ACCs (F(1,20) = 0.070, p = 0.794 for RTs, F(1,20) = 2.892, p = 0.108 for ACCs respectively) indicating that the time and accuracy for left hand and right hand stimuli responses were comparable. The ANGLE effect on RTs was significant (F(5, 100) = 12.434, p<0.001) and RT increased with the angle from the upright position (0°) and reached the maximum at 180° (Figure 2C), and ACC correspondingly decreased with angle and reached the minimum at 180° (ANGLE on ACCs: F(5, 100) = 15.799, p<0.001, Figure 2D), indicating a significant “angle effect” on behavior performance. Significant ANGLE × GROUP interaction effect was observed on ACCs (F(5,100) = 3.599, p = 0.005). Follow-up tests showed stroke patients had significantly lower ACC particularly at 180° (p<0.001). Similar to those reported results, symmetric angles didn’t show significant differences of RT or ACC between 60° and 300°, 120° and 240° for either control subjects or stroke patients (all p>0.05). Therefore, the angles were merged into four categories: 0°, ±60° (for 60° and 300°), ±120° (for 120°and 240°) and 180° hereafter as shown in Figure 2C and D. In short, both groups could complete MRT, significant impairment of behavior performance with longer RT and lower ACC was observed for stroke patients.

### Electrophysiological Results

In order to understand the details of the brain activations during MRT, both phase-locked ERP (i.e., P200 and P300) and phase-unlocked ERD/ERS in beta band in each sub-stage were investigated. Three within-subjects factors, i.e., HAND, ANGLE and REGION (13 brain areas as shown in Figure 1B) were considered in ANOVA of the amplitudes of P200, P300 and the ERD/ERS indexes in three sub-stages (i.e., Beginning, Middle and End).

#### Visual perceptual cognitive process (Beginning sub-stage)

**Figure 3:**
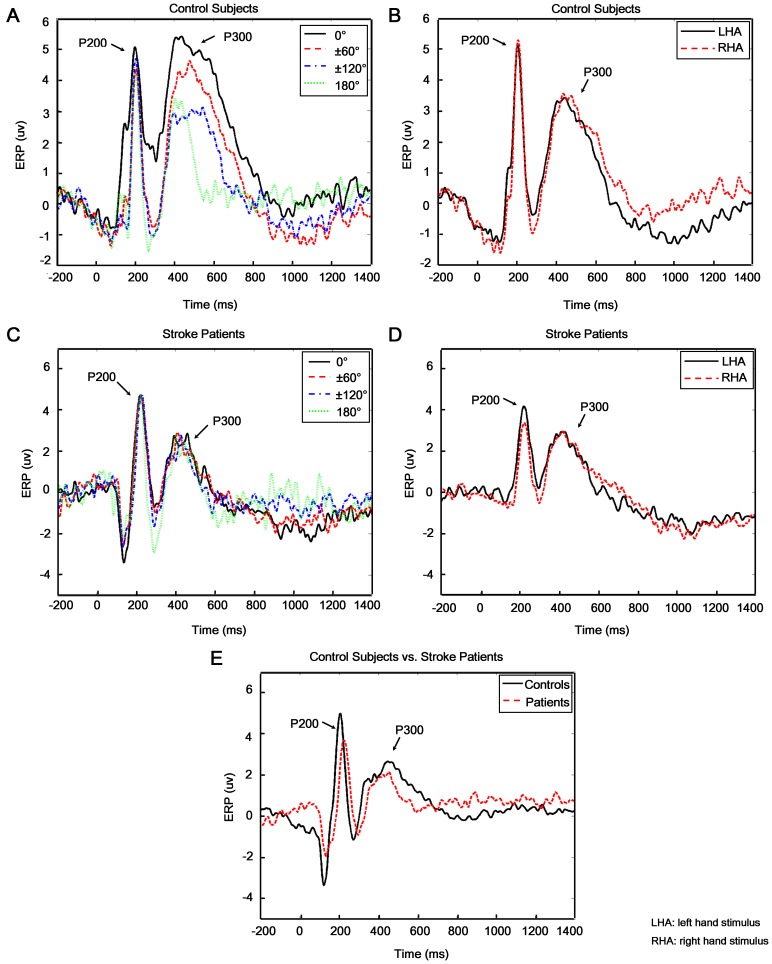
Event-related potential (ERP) results. **A** and **B:** Grand-averaged ERP curves at P4 electrode for control subjects were shown in regard to ANGLE and HAND factors respectively. **C** and **D:** ERP curves at P4 electrode for stroke patients also with respect to ANGLE and Hand factors respectively. **E:** Comparison of grand-averaged ERP at P4 electrode between control subjects and stroke patients was shown. Stimuli were displayed on monitor at 0 ms. Baseline was from −200 ms to 0 ms, and two significant important components, i.e., P200 (0–300 ms) and P300 (300–800 ms) were indicated by arrows. No significant difference of P300 amplitude between 60° vs. 300° (p = 0.384), 120° vs. 240° (p = 0.293) was observed for all subjects. Thus only four ERP curves of 0°, ±60°, ±120° and 180° were illustrated in A and C.

**Figure 4:**
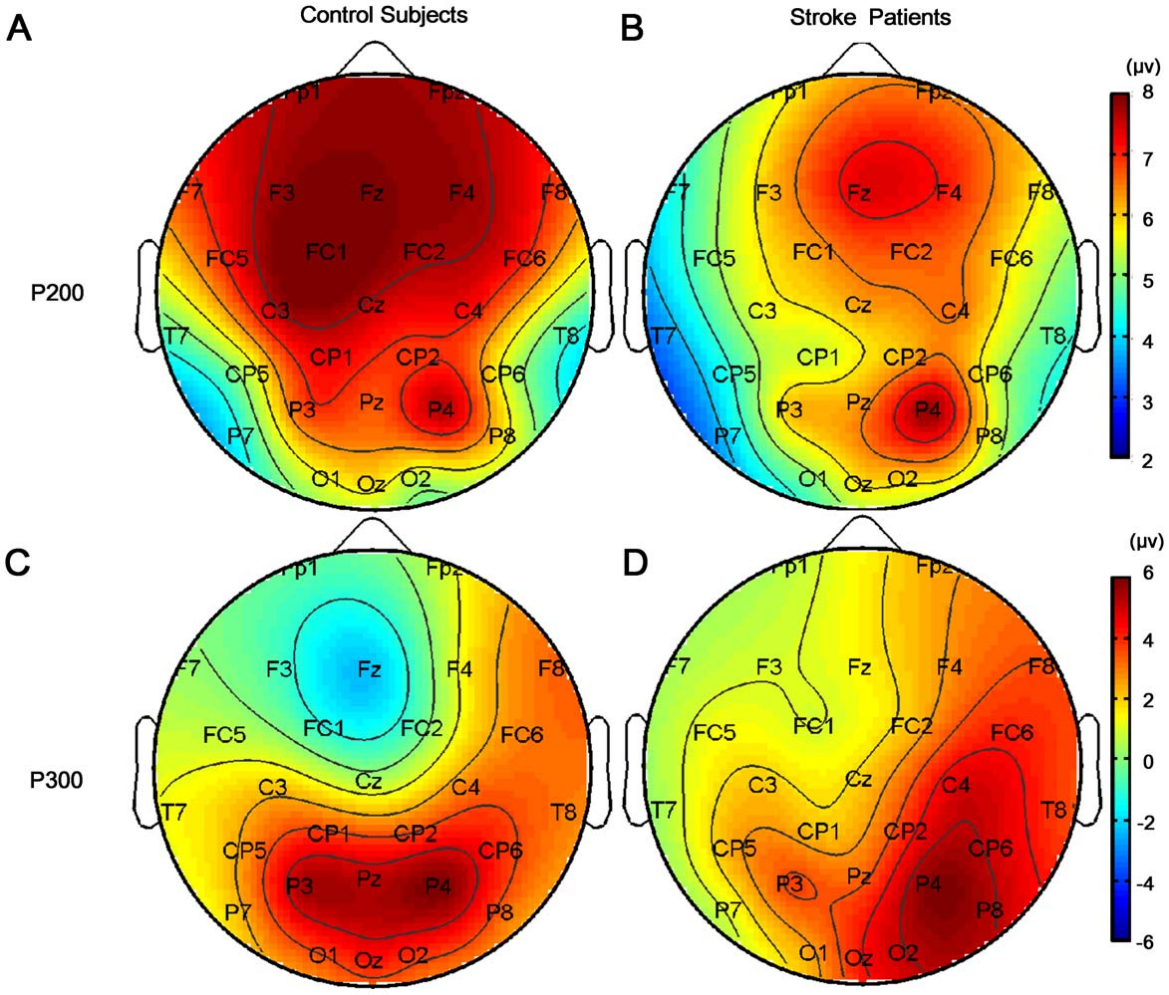
Brain mappings of ERP. **A** and **C:** Brain mappings of P200 and P300 for control subjects were shown respectively. **B** and **D:** Brain mappings of P200 and P300 for stroke patients were illustrated. In the brain mappings of ERP, more red color represented higher phase-locked activation.

No significant differences in amplitude of early ERP component (i.e., P200) was found between stroke patients and control subjects (GROUP effect: F(1,20) = 0.354, p = 0.559) (Figure 3E). The insignificance of ANGLE (F(5,100) = 1.074, p = 0.379) and HAND (F(1,20) = 0.240, p = 0.629) effects indicated that the phase-locked brain activation for left hand and right hand rotated to different angles were comparable in early cognitive process (Figure 3A and B for control subjects; Figure 3C and D for stroke patients). However, significant REGION effect and its interaction with GROUP on P200 amplitude was observed (REGION: F(12,240) = 2.094, p = 0.018; REGION × GRROUP: F(12,240) = 1.643, p<0.001). Brain mappings of P200 were illustrated in Figure 4A for control subjects and B for stroke patients. Both control subjects and stroke patients showed prominent phase-locked activation of P200 component in the right parietal lobe (P4). Moreover, larger brain areas in frontal and central lobe in control subjects were observed with prominent activation of P200 which was found only in the right frontal lobe (Fz and F4) in stroke patients. Hypo-activation in frontal and central lobe in ipsilesional (left) hemisphere might imply the impairment of early visual perceptual cognitive process after stroke.

**Figure 5:**
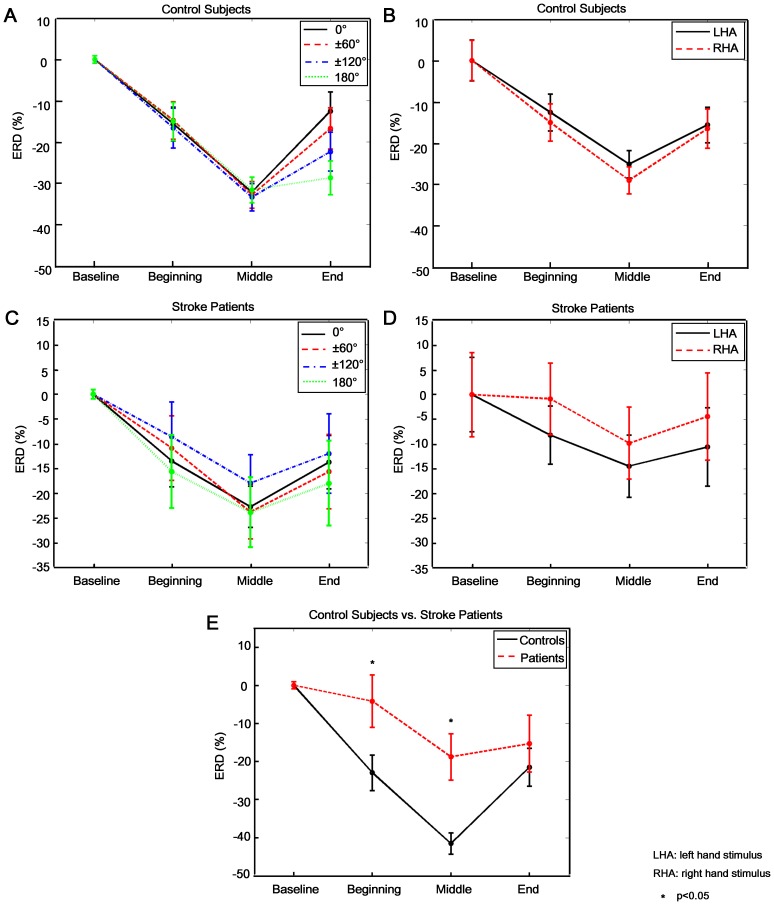
Event-related desynchronization in beta band (beta-ERD). **A** and **B:** Beta-ERD lines at Pz electrode for control subjects were illustrated with respect to ANGLE and HAND factors respectively. **C** and **D:** Beta-ERD lines at Pz electrode for stroke patients in regard to ANGLE and HAND factor respectively were shown. **E:** The comparison of beta-ERD between control subjects and stroke patients was illustrated. All ERD/ERS indexes were based on normalization with Baseline (i.e., ERD/ERS index in its corresponding sub-stage minus Baseline ERD/ERS index), thus ERD/ERS index in Baseline was 0%. And, ERS/ERD indexes were negative in other three sub-stages, thus significant ERD was observed in all subjects. Similar with ERP results, no significant difference of beta-ERD between 60° vs. 300° (p = 0.278), 120° vs. 240° (p = 0.352) was observed for all subjects. Thus only four beta-ERD lines of 0°, ±60°, ±120° and 180° were illustrated in A and C.

**Figure 6:**
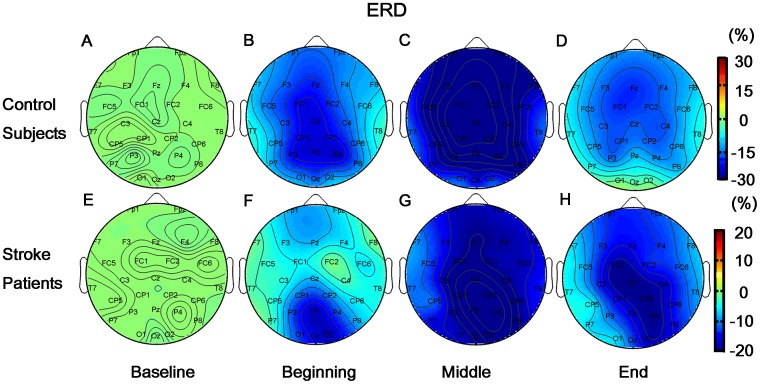
Brain mappings of beta-ERD. **A–D:** Beta-ERD mapping results for control subjects were shown in four sub-stages. **E–H:** Beta-ERD mapping results for stroke patients were illustrated which were corresponding to four sub-stages respectively. All other three sub-stages had lower ERD than Baseline, and deeper blue color represented higher phase-unlocked activation (i.e., more negative compared with Baseline).

In contrast to the ERP results, beta-ERD values in this sub-stage showed significant effect of GROUP (F(1,20) = 8.460, p = 0.009), i.e., stroke patients had significantly lower beta-ERD than the controls (Figure 5E). The insignificance of ANGLE (F(5,100) = 0.556, p = 0.733) and HAND (F(1,20) = 0.903, p = 0.353) was due to phase-unlocked brain activation for left hand and right hand stimuli with different angles were comparable (Figure 5A and B for control subjects; C and D for stroke patients). But significant REGION effect and its interaction with GROUP were observed (REGION: F(12,240) = 7.268, p<0.001; REGION × GRROUP: F(12,240) = 4.639, p<0.001) (Figure 6B for control subjects and F for stroke patients). Major ERD was observed in precentral (FC1 and FC2), central (Cz, CP1 and CP2) and parietal area (P3, Pz and P4) in control subjects. However, stroke patients only showed major ERD in parietal (P3, Pz and P4) and occipital (O1, Oz and O2) area. In contrast to control subjects, patients showed less movement-related cortical area (frontal-central) and more visual cortical (occipital) activations.

During early visual perceptual cognitive process, neither group showed HAND or ANGLE effect in ERP or ERD/ERS. Prominent hypo-activation in motor-related frontal and central areas was observed in stroke patients, which might lead to the deficiency in hand perception and spatial information encoding in stroke patients.

#### Mental rotation cognitive process (Middle sub-stage)

The mental rotation sub-stage was investigated by looking into the amplitude of P300 and beta-ERD. Similar to the P200 in early sub-stage, GROUP effect of P300 was not significant either (F(1,20) = 0.208, p = 0.654) in Middle sub-stage. The insignificance of main effect of HAND (F(1,20) = 3.046, p = 0.096; Figure 3B for control subjects and D for stroke patients) illustrated that the phase-locked activation during mentally rotating of left and right hand stimulus was comparable. The ANGLE factor was significant (F(5,100) = 2.390, p = 0.043), i.e., P300 amplitudes for different angles were significantly different. The ANGLE × GROUP interaction was also significant (F(5,100) = 3.319, p = 0.008). Follow-up tests showed that P300 amplitude in control group decreased as the angle increased from upright position (0°) to 180°, indicating significant mental rotation phenomena (p<0.002), which was called “amplitude modulation effect” (Figure 3A). However, such an effect was not observed in stroke patients (p>0.058) (Figure 3C). The significant effect of REGION and its interaction with GROUP (REGION: F(12,240) = 8.071, p<0.001; REGION × GROUP: F(12,240) = 9.789, p<0.001) were due to the fact that phase-locked brain activation for stroke patients was significantly different from control subjects. Significant P300 activation was observed in parietal lobes in both hemispheres (P3, Pz and P4) for control subjects (Figure 4C), which was observed only in the right parietal lobe (P4, P8 and CP6) in contralesional hemisphere for stroke patients, indicating significant “lateralization” effect of P300 (Figure 4D). In short, hypoactive phase-locked activation in ipsilesional parietal lobe possibly implied the impaired cognitive process of spatial information processing and mental rotation for stroke patients.

Further analysis of phase-unlocked activity showed beta-ERD in most brain areas in all subjects during mental rotation sub-stage. In particular, stroke patients had significantly lower beta-ERD than the control subjects (GROUP effect: F(1,20) = 7.550, p = 0.012) (Figure 5E). The insignificance of ANGLE (F(5,100) = 1.345, p = 0.252) and HAND (F(1,20) = 0.069, p = 0.796) effect indicated that phase-unlocked brain activation for left hand and right hand at each angle was comparable (Figure 5A and B for control subjects; C and D for stroke patients). Significant REGION effect on beta-ERD (F(12,240) = 14.110, p<0.001) and its interaction with GROUP (F(12,240) = 5.571, p<0.001) was observed, as shown in Figure 6 (C for control subjects and G for stroke patients). An interesting finding was that FC5 and CP5 loci in stroke patients showed significantly weaker ERD than other areas. Overall, stroke patients showed hypoactive motor-related brain areas in ipsilesional hemisphere during the mental rotation sub-stage.

In Middle sub-stage of MRT, lateralization of P300 in parietal lobe and beta-ERD in ipsilesional movement-related cortex might reflect poor spatial information processing and mental rotation ability in stroke patients.

#### Response cognitive process (End sub-stage)

Then we look at the end stage of MRT, i.e., the cognitive process starting at 800 ms post stimulus. Since the phase-locked activations in such a later period were more complicated, it’s not practical to analyze the ERP components. We only analyzed the phase-unlocked ERD/ERS in the End sub-stage.

**Figure 7:**
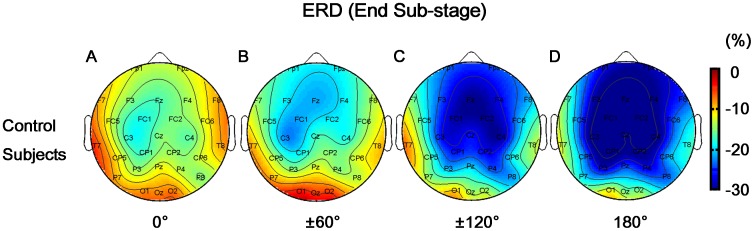
Brain mappings of beta-ERD for control group at different angles in response sub-stage. Higher ERD in larger brain areas particularly in right hemisphere was found with larger angles in control subjects (i.e., more brain areas with deeper blue color represented higher phase-unlocked cortical activation).

Main effect of GROUP on beta-ERD was not observed (F(1,20) = 2.272, p = 0.147) (Figure 5E). Significant effect of REGION (F(12,240) = 13.425, p<0.001) and its interaction with GROUP (F(12,240) = 2.746, p = 0.002) were found. Most frontal-central areas (F3, Fz, F4, FC1, FC2, C3, Cz, C4, CP1 and CP2) in control subjects showed significant beta-ERD (Figure 6D). In contrast, stroke patients showed less brain areas (FC1, Cz, CP2, P4 and O2) with beta-ERD (Figure 6H). In addition, significant HAND effect (F(1,20) = 4.863, p = 0.039) and its interaction with GROUP (F(1,20) = 2.141, p = 0.046) were also observed. Specifically, stroke patients showed overall lower beta-ERD for right hand (affected) than that for left hand (p = 0.018) (Figure 5D). However, beta-ERD in control group was independent of the stimulus parity of HAND (p = 0.190, Figure 5B). Further, both main effect of ANGLE (F(5,100) = 3.916, p = 0.003) and its interaction with GROUP were significant (F(5,100) = 2.653, p = 0.027). Interestingly, “angle effect” on beta-ERD was only found in control subjects (Figure 5A), i.e., higher beta-ERD values in more brain areas for larger spatial angles, particularly in the right hemisphere (Figure 7). However, such an effect was not observed in stroke patients (Figure 5C), which might be due to the impairment of the spatial information processing.

In short, in contrast to the controls, stroke patients showed significant dependence of beta-ERD on stimulus parity of HAND, and the absence of “angle effect” in the End sub-stage of MRT.

## Discussion

The present study investigated the spatiotemporal alternations of the cognitive process in each sub-stage during MRT by means of behavior performance, ERP and beta-ERD. Both behavior and electrophysiological results showed that the motor imagery cognitive process in stroke patients was significantly impaired when compared with the age-matched control subjects. In behavior, stroke patients presented significantly lower accuracy rate and slower response to stimulus pictures of both affected and unaffected hands. In electrophysiology, ischemic lesion led to hypo-activations in frontal and central areas in the aspects of both P200 and beta-ERD during early stimuli perception, followed with a lateralized parietal P300 and a spreading lower beta-ERD in the subsequent mental rotation sub-stage. Particularly, stroke patients didn’t show any “angle effect” as in health subjects during the response execution. We will discuss these results from the following perspectives (i) behavior impairment, (ii) hypo-activation in visual perceptual cognitive process, (iii) lateralization in mental rotation sub-stage and (iv) loss of “angle effect” in response process.

### Behavior Impairment

Both stroke patients and control subjects showed the “angle effect” in behavior. Stroke resulted in longer RT and lower ACC in patients. It was noted that responses to both affected (right) and unaffected hands were significantly impaired after left hemispheric stroke. It was proposed that the motor areas of both hemispheres were functionally coupled and equally balanced in terms of “mutual inhibitory control” in health subjects. It has been documented that normal hand movement was associated with enhanced activation in contralateral motor areas and the inhibition toward its counterpart areas in the ipsilateral hemisphere [50]. In addition, the left hemispheric motor area lesion could lead to bilateral movement disorders, whereas right hemispheric stroke was more likely to have only unilateral (contralateral) motor deficits [51], [52], showing a “left hemispheric dominance” effect in movement. Thus, the prolonged response to unaffected hands (left) might be due to the reduced inhibitory interaction from the right hemisphere to its left counterpart after stroke. Furthermore, the behavior results of the stroke patients showed a similar “left hemispheric dominance” effect in motor imagery task, indicating mental and actual movements might share some similar neural substrates. More data of the right hemispheric stroke patients in motor imagery are needed to further understand the underlying mechanisms.

### Hypo-activation during Visual Perceptual Cognitive Process

Previous studies of MRT with hand stimuli on healthy subjects have also shown significant motor areas activations (e.g., BA6 and M1), which implied the important role of motor cortex during mental rotation cognition [53]. Furthermore, neuroimaging studies with event-related fMRI revealed that MRT could activate dorsolateral premotor cortex in the early perception encoding of visual stimuli, which also confirmed the direct involvement of the motor-related areas activation in this sub-stage [54]. However, stroke subjects presented hypoactive P200 prominently in the ipsilesional motor-related central-parietal cortex and decreased bilateral frontal ERD. We speculated that stroke patients’ cortex was mainly engaged in encoding the physical properties of hand pictures (e.g., color, shape etc.) in this sub-stage, showing activations prominently in the occipital and parietal cortex for visual information processing. The loss of activation in the hand movement related areas in patients group could be due to the impaired movement planning after the stroke. In addition, the hypo-activation in motor cortex in ipsilesional hemisphere might consequently impair the visual and motor information integration for visual information processing and preparation for the following mental movement sub-stage, such integration was shown in control subjects during early visual perceptual cognitive process.

### Lateralization during Mental Rotation Cognitive Process

Significant activations in the parietal cortex, premotor cortex and dorsolateral prefrontal cortex during the whole stimulus presentation period was observed in previous fMRI study of MRT on healthy subjects, indicating the network was similar with actual movement [55]. Our results for controls also showed activations in parietal (P300) and motor-related areas (ERD) in mental rotation sub-stage. However, in contrast to controls, stroke patients showed lateralization effect of P300, i.e., P300 was significantly lower in ipsilesional parietal lobe than its counterpart; and hypoactive motor-related cortex, i.e., the ERD was significantly lower in premotor cortex and dorsolateral prefrontal cortex after stroke. Parietal cortex, particular in the left hemisphere, was responsible for movement-related spatial perception, e.g., sensory of hand position and motion status, and movement planning [56]–[58]. In this study, we actually didn’t find a significant change of P300 in the contralesional parietal cortex in stroke patients when compared with control subjects. Therefore, the lateralization effect after stroke was mainly due to the impairment of the spatial information processing network in the left parietal cortex. Furthermore, previous studies also showed that frontal motor-related areas, e.g., premotor cortex, played a central role in the selection of movements [57]. In this study, frontal hypo-activation in stroke patients indicated their deficiency in parity judgment and the subsequent movement execution. Thus, the P300 lateralization and hypoactive motor-related cortex during mental rotation sub-stage closely correlated with the poor ability to process spatial information and motor planning in stroke patients.

### Loss of “Angle Effect” during Response Cognitive Process

For control subjects, task-difficulty-dependent ERD value and area were found in both hemispheres, particularly in the right (i.e., “angle effect”), which was in line with previous results that the right hemisphere was more activated during a more difficult task [59], [60]. However, the cognitive process in all sub-stages in this study did not show significant “angle effect” on either ERP or beta-ERD for stroke patients. Such a cognitive impairment after stroke might be due to the injury of the spatial information processing pathway to the posterior parietal cortex in ipsilesional hemisphere. In control group, the beta-ERD in response sub-stage was limited in frontal and central cortex. In contrast, stroke patients showed cortical activation at right occipital and parietal area in the End sub-stage, which might be due to the delayed or prolonged visual information encoding or mental rotation sub-stages after stroke. The loss of “angle effect” in stroke group also manifested hypo-activation in the ipsilesional precentral area which was considered to be related with the judgment, planning and coordination of complex hand movements [61]–[64]. Hypo-activation in these brain areas might correlate with the poor judgment and execution for stroke patients in response sub-stage.

### Study Limitation

This study was based on the data collected from only eleven stroke patients. All subjects were selected with motor function impairment due to the stroke in left hemisphere, nevertheless, we didn’t consider the variance of the lesion location, e.g., parietal lobe, frontal lobe etc. In order to know the roles of a specific cortex in the cognitive process of MRT in stroke patients, larger sample size with more stringent restrictions of lesion locations are recommended. In addition, studies on other EEG bands, e.g., alpha or gamma, could offer new insights into the cognitive process of motor imagery. Furthermore, combining EEG with fMRI could provide high spatiotemporal resolution of structural and functional information in deep brain, which may show more details of the cognitive process of motor imagery after stroke.

### Conclusion

In summary, we investigated the cortical alternations in each cognitive sub-stage during MRT after the left hemispheric stroke injury using both behavior and electrophysiological methods. Movement impairment after stroke would also accompanied by poor behavior performances in motor imagery. Brain mappings of ERP and beta-ERD showed hemispheric lateralization after stroke due to the ipsilesional hypo-activation during visual stimuli perception and mental rotation. Furthermore, stroke lesion also resulted in the loss of “angle effect” due to the impaired spatial information processing during response execution. In conclusion, stroke lesion altered the brain activation in each sub-stage of motor imagery. The results might provide new insights into the understanding of cognitive process during mental rotation, and could be referenced as a guide in stroke rehabilitation with motor imagery training.
